# Selective Nonoperative Management of a Pediatric Abdominal Gunshot Wound

**DOI:** 10.7759/cureus.85575

**Published:** 2025-06-08

**Authors:** David M Dodson, Aashish Rajesh, Christopher Brown, Mark T Muir

**Affiliations:** 1 Department of Surgery, The University of Texas Health Science Center at San Antonio, San Antonio, USA

**Keywords:** angioembolization, pediatric trauma care, penetrating abdominal trauma, penetrating liver trauma, selective nonoperative management

## Abstract

The standard of care for penetrating abdominal trauma (PAT) has traditionally been exploratory laparotomy. However, significant rates of surgical morbidity and nontherapeutic laparotomies have prompted the development of alternative strategies. Selective nonoperative management (SNOM) is one such approach, which can be considered for hemodynamically stable patients without signs of peritonitis. We present the case of a 17-year-old male patient who sustained a gunshot wound (GSW) to the upper abdomen and right flank. Imaging revealed a grade IV liver laceration with active extravasation. The patient was admitted for serial abdominal exams, hematologic monitoring, and a scheduled computed tomography (CT) angiography in 72 hours. Interventional radiology performed embolization of multiple hepatic artery pseudoaneurysms on hospital day 4 based on the CT angiography findings. The patient was transitioned to a regular diet and was discharged without complication on hospital day 6. This case highlights the safety and efficacy of CT-guided SNOM and delayed angioembolization in pediatric patients with PAT.

## Introduction

Exploratory laparotomy has long been the standard approach for transperitoneal penetrating abdominal trauma (PAT) [1]. However, growing evidence highlights the drawbacks of this practice, notably a 10-15% rate of nontherapeutic laparotomies and their associated complications [2,3]. A National Trauma Data Bank (NTDB) analysis (2007-2019) reported a 12% nontherapeutic laparotomy rate in blunt trauma, with higher crude mortality (31.5% vs 20.5%) and a 1.33-fold increased adjusted risk of death compared to therapeutic laparotomy [4]. 

While selective nonoperative management (SNOM) for PAT was once controversial, Peponis et al. demonstrated its potential feasibility and benefits, including reduced complications, shorter hospital stays, and lower mortality, in adult patients across level I and II trauma centers [5]. However, data on pediatric PAT remain limited. Children’s unique physiology makes signs of deterioration, such as hypotension or worsening abdominal exams, more subtle and delayed. A 2009 study by Cigdem et al. reported that of 90 children with PAT, 56% were managed without surgery, with only two requiring therapeutic laparotomy within 24 hours [6]. We present the role of computed tomography (CT) in mapping the trajectory of PAT in children and its utility in guiding safe SNOM.

## Case presentation

**Figure 1:**
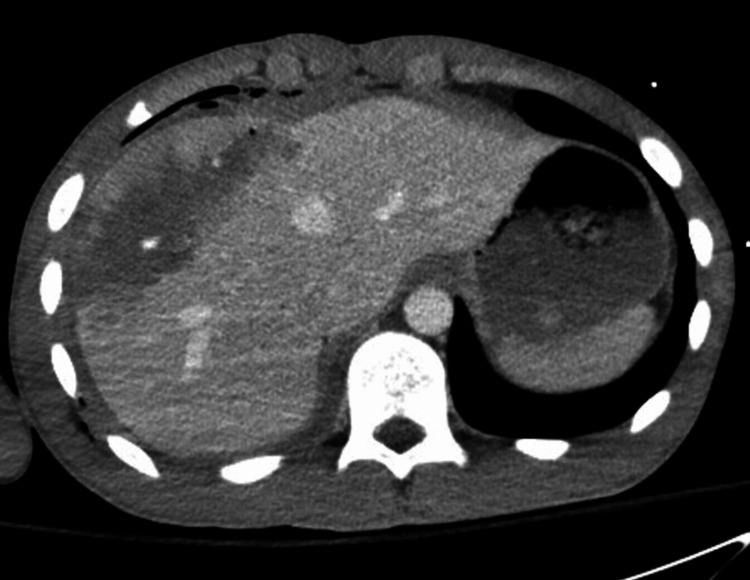
Computed tomography (CT) scan on hospital day 1 showing a grade IV liver laceration with active extravasation

A previously healthy 17-year-old male patient presented to our level I trauma center following a gunshot wound (GSW) to the upper abdomen. He was alert, hemodynamically stable, and exhibited focal midepigastric tenderness. A second GSW was observed at the right flank near the eighth rib. Contrast CT revealed a grade IV liver laceration with active contrast extravasation, a small perihepatic hematoma, moderate hemoperitoneum, and a small pneumothorax (Figure 1).

Labs showed a hemoglobin of 13.9 g/dL and elevated transaminases. The patient was admitted to the pediatric intensive care unit for serial labs and monitoring (Table 1).

**Table 1:** Lab trends AST: Aspartate aminotransferase (normal reference range 8-36 U/L); ALT: Alanine aminotransferase (normal reference range 4-36 U/L); Hb: Hemoglobin (normal reference range 11.5-14.9 g/dL)

Hospital Day	AST (U/L)	ALT (U/L)	Hb (g/dL)
1	136	134	14.1
2	229	223	13.1
3	567	623	12
4	293	453	8.3
5	168	340	9.1

**Figure 2:**
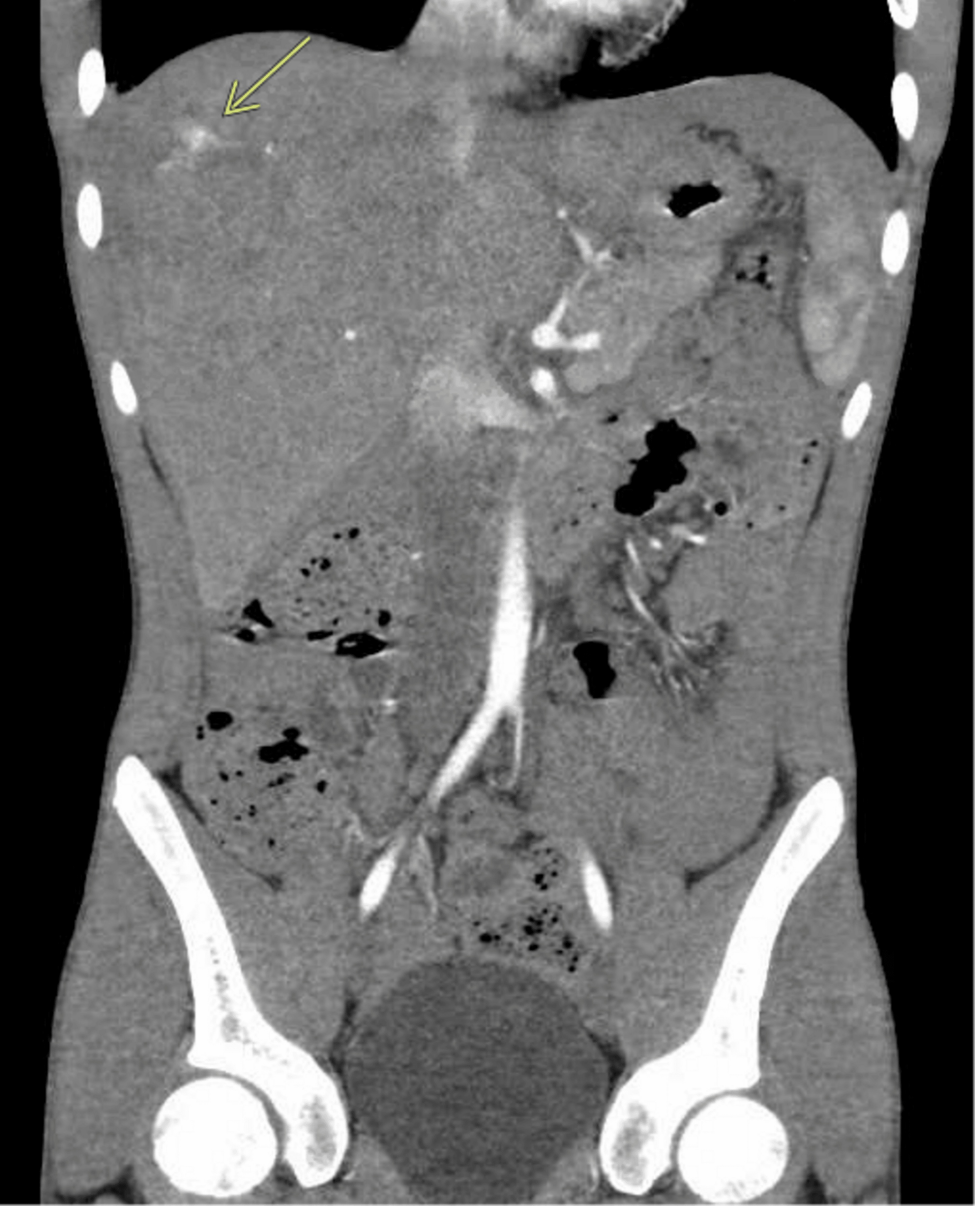
Computed tomography (CT) angiography scan on hospital day 4 demonstrating pseudoaneurysms and active arterial extravasation (arrow)

The patient's hemodynamics, including heart rate and blood pressure, remained normal throughout his clinical course. A drop in hemoglobin from 12 g/dL to 8.3 g/dL on hospital day 4 prompted CT angiography, which identified pseudoaneurysms in hepatic segments 4A/4B and 8 (Figure 2). These were successfully embolized with Gelfoam (Pfizer Inc., New York, USA). The patient remained stable post-procedure and was discharged on hospital day 6. He was noted to be doing well when seen in clinic two weeks following discharge, and required no additional follow-up imaging.

## Discussion

This case highlights two important considerations in the management of penetrating abdominal trauma in pediatric patients. Firstly, CT-guided SNOM is a viable approach for pediatric patients with right upper quadrant injuries involving the liver, particularly when the trajectory spares hollow viscera and the patient remains hemodynamically stable without signs of peritonitis. Secondly, immediate angioembolization is not universally required, even in the presence of active contrast extravasation from solid organ injury, provided the patient maintains clinical stability.

Our findings are consistent with those of Sakamoto et al. (2018), who analyzed data from over 3,000 pediatric patients with penetrating solid organ injuries in the NTDB. SNOM was attempted in 20.5% of cases, with an overall success rate of 71.5%, and liver injuries demonstrating the highest success rate at 80.5% [7]. Predictors of SNOM failure included hemodynamic instability, GSW mechanism, and associated hollow viscus injuries. At our trauma center, it is standard practice to obtain routine CT angiography approximately 72 hours after injury in adult patients with grade III or higher solid organ injuries. In pediatric patients, however, our decision to pursue follow-up imaging is guided by clinical status and laboratory trends. While angioembolization is widely employed in adult trauma patients with arterial contrast extravasation, the American Pediatric Surgical Association (APSA) guidelines recommend its use in children only when there are ongoing clinical signs of bleeding after initial resuscitation [8].

As evidence continues to evolve in support of selective nonoperative strategies for penetrating abdominal trauma, it is important to revisit the Eastern Association for the Surgery of Trauma (EAST) guidelines. The 2010 EAST recommendations offer a level III endorsement, reflecting limited but suggestive evidence, for nonoperative management in cases of isolated right upper quadrant PAT in hemodynamically stable patients with reliable clinical examinations and minimal abdominal tenderness [3]. The role of laparoscopy in the management of PAT remains an area of active investigation. Recent meta-analyses suggest that, in stable patients, laparoscopy is associated with fewer complications and a shorter hospital stay compared to nontherapeutic laparotomy [9,10]. Ultimately, the decision to pursue operative versus nonoperative management (and when operative, to choose laparoscopy over laparotomy) should be informed by cross-sectional imaging, the clinical expertise of the surgical team, and the institution’s capacity for continuous monitoring to ensure timely detection of clinical deterioration and minimize delays in intervention.

An additional consideration is the definition of the pediatric age group. While the American College of Surgeons Committee on Trauma defines a level I pediatric trauma center as one that provides specialized care to at least 200 children under the age of 15 annually [11], definitions of "pediatric" vary across institutions, with some using cutoffs of 16 or 18 years. At our institution, all patients under the age of 18 are managed by a dedicated pediatric trauma team. This model ensures that adolescents, including older teenagers, receive developmentally appropriate, specialized care and benefit from robust injury prevention education and pediatric-specific resources, ultimately supporting optimal outcomes in this unique patient population.

## Conclusions

In summary, this case highlights a key shift in pediatric trauma care: CT imaging can accurately map injury trajectory to support SNOM in stable pediatric patients. Imaging-guided close monitoring and embolization in the setting of clinical deterioration can help avoid unnecessary laparotomies. For surgeons managing pediatric trauma, these images underscore a modern paradigm: in the right patient, restraint, guided by imaging, can be just as lifesaving as the scalpel.
